# The Train-Line Pattern on Duplex Ultrasound Helps Differentiate Intramural Thrombus from Atheromatous Plaque in Common Carotid Artery Dissection

**DOI:** 10.3390/diagnostics15101297

**Published:** 2025-05-21

**Authors:** Ming-Hsing Chang, Yen-Yu Huang, Fang-I Hsieh, Kuan-Yu Lin, Hsu-Ling Yeh, Kai-Jing Yeh, Li-Ming Lien

**Affiliations:** 1 Department of Neurology, Shin Kong Wu Ho-Su Memorial Hospital, Taipei 111045, Taiwan; neurotttttttt@gmail.com (M.-H.C.); alexlingy0315@hotmail.com (K.-Y.L.); mirage.yeh@gmail.com (H.-L.Y.); yehs999888@gmail.com (K.-J.Y.); 2 Chen Sen-Feng United Clinic, Taipei 114002, Taiwan; kikilaa@hotmail.com; 3 School of Public Health, College of Public Health, Taipei Medical University, Taipei 110301, Taiwan; hsiehfangi@tmu.edu.tw; 4 College of Medicine, Taipei Medical University, Taipei 110301, Taiwan

**Keywords:** train-line pattern, common carotid artery dissection, carotid duplex sonography, aortic dissection, stroke diagnosis, neuroimaging

## Abstract

**Background/Objectives:** Common carotid artery dissection (CCAD) can result in severe neurological sequelae; however, its diagnosis may be challenging due to consciousness disturbance and aphasia. The objective of this article is to propose a new imaging feature to assist in the identification of CCAD. **Methods**: This retrospective case series enrolled 139 patients with discharge diagnoses of aortic dissection who underwent carotid ultrasound during admission over a period of three years. **Results**: Among these patients, 23 had type A aortic dissection, and 113 had type B aortic dissection or related conditions. Notably, among the 23 patients with type A aortic dissection, eight had associated common carotid artery dissection (CCAD), and a total of nine CCAD events were identified. Meanwhile, a B-mode ultrasound revealed six double lumens with intimal flaps and three intramural thrombi. The ‘train-line’ pattern in ultrasonography was discerned by detecting a hypoechoic thickened wall, which was characterized by a margin formed by two parallel linear reflections in close proximity. This distinctive “train-line” pattern was identified in three intimal flaps and two intramural thrombi. **Conclusions**: While double lumens and intramural thrombus are prevalent findings, the latter may be misinterpreted as atherosclerotic plaque. The “train-line” pattern may aid in distinguishing intramural thrombus from atheromatous plaque, offering an additional diagnostic tool alongside the identification of double lumens with intimal flaps.

## 1. Introduction

Aortic dissection Stanford type A includes DeBakey type I and type II, affecting either both the ascending and descending aorta or the ascending aorta alone [1]. The tearing of the aortic media and intima leads to development of the dissection [2,3]. Aortic dissection type A accounts for 70–75% of all aortic dissections [3], and it is associated with high mortality and unfavorable outcomes [3,4]. In patients with aortic dissection type A, the mortality rate for those who undergo surgery is 26%, and the mortality rate for those who receive medical treatment alone without surgery is 58% [5]. Twenty-four percent of aortic dissection would extend to the common carotid artery (CCA), increasing the risk of stroke [6]. The diagnosis is highly dependent on clinical suspicion [4]. Common carotid artery dissection (CCAD) often manifests with neurological symptoms such as syncope, impaired consciousness, language dysfunction, headaches or neck pain, blindness in one eye, and hemiparesis [2,7]. Furthermore, CCA malperfusion is associated with postoperative stroke and prolonged stays in the intensive care unit and hospital [8]. However, an altered state of consciousness or aphasia may obscure the underlying aortic dissection and delay the diagnosis in stroke patients. Not being rare, the incidence of transient or permanent neurologic symptoms in patients with aortic dissection reaches up to 42%, of which the cerebrovascular events range from 6.1 to 32% [5,9]. Considering the high proportion of neurological symptoms, it is crucial to timely and accurately identify CCA dissection.

Cervical carotid dissection must be differentiated from other factors that cause thickening of the arterial wall, including radiation therapy, vasculitis, and atherosclerosis [10]. Prompt carotid ultrasonography performed in the emergency department represents a practical and effective diagnostic tool for detecting CCAD, which may serve as an important indicator of underlying aortic dissection. Several sonographically pathologic structures of dissection were addressed in previous studies. The most characteristic finding in the carotid duplex for dissection is a double lumen with a free flap [10,11]. However, an intimal flap floating in either the true or false lumen is rarely perceived [10]. Other B mode findings include intramural thrombus or hematoma, occlusion or severe stenosis, pseudoaneurysm, and smooth or tapering vascular lumen [7,10,12,13]. The dissection-related intramural thrombus is presented in around one third of patients with CCAD, but it is hard to distinguish from an aging-related atherosclerotic thrombus [10,11]. We noticed the “train-line” pattern of intramural thrombus through observations in our patients, and it could help differentiate these two situations.

## 2. Materials and Methods

We conducted the retrospective case series by searching the medical database of the Shin Kong Wu Ho-Su Memorial Hospital, which is a tertiary-centered hospital in Taipei, Taiwan. Patients who had aortic dissection listed in their discharge diagnosis and carotid ultrasound report were enrolled retrospectively over a period of three years. A total of 139 patients were recruited into our study.

The patients were investigated with a Philips (Bothell, WA, USA) ultrasound system (CX50) equipped with a 12–3 MHz phased linear array transducer probe. The procedures of carotid duplex sonography were performed by well-trained technicians. The patient lay on an examination bed in a supine position, while the technician sat at the patient’s superior cranial side. To maximize exposure of the ipsilateral neck, the patient’s head is rotated 45 degrees to the contralateral side. In addition, the patient should remain in a relaxed state to prevent unclear sonographic images. By placing a transducer probe on the lower part of the neck, we evaluated the echotomographies of CCA via real-time B-mode imaging, color Doppler, and pulsed Doppler. Sonographic images were saved on the CX50 and uploaded to the medical information system of Shin Kong Wu Ho-Su Memorial Hospital. Information on the CCA, including peak systolic velocity, end diastolic velocity, resistance index, and blood flow, was collected.

We reviewed every medical file and image by two clinicians. The diagnosis of aorta and branch artery dissection would be confirmed by contrast computed tomography (CT) alone or by both CT and operation findings. The presence of ischemic stroke would be verified by brain magnetic resonance imaging (MRI). Information on in-hospital mortality was collected. The reports were interpreted by competent neurologists.

The “train-line” pattern on ultrasonography was detected, which was characterized by a margin consisting of two hyperechoic parallel linear reflections that are closely positioned and override a thickened wall. It was identified subjectively by neurologists specializing in neurosonography. To evaluate inter-observer agreement in identifying the “train-line” pattern, we used Cohen’s Kappa (κ), which is a statistical measure that quantifies the degree of agreement between two raters classifying items into categorical outcomes. A total of twelve representative carotid ultrasound images were selected, encompassing the following five categories: atherosclerotic plaque, double lumen with the train-line pattern, double lumen without the train-line pattern, intramural thrombus with the train-line pattern, and intramural thrombus without the train-line pattern. Two experienced neurologists specializing in neurosonography independently reviewed and categorized the images. The calculated Cohen’s Kappa value (standard error) was 0.824 (0.169), indicating almost perfect agreement (κ > 0.8). The study program was approved by the Institutional Review Board of Shin Kong Wu Ho-Su Memorial Hospital.

## 3. Results

**Figure 1:**
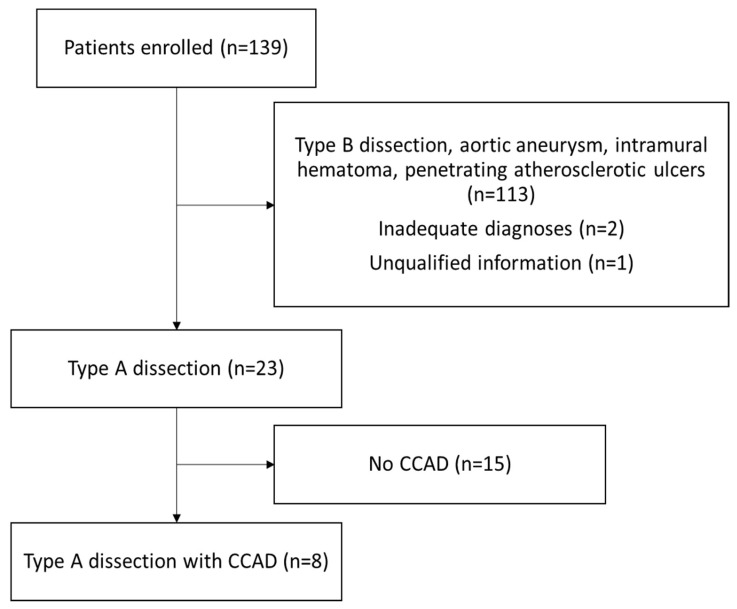
Study design flow chart of patients enrolled and reasons for exclusion. **Abbreviation:** CCAD, common carotid artery dissection.

Among the 139 patients enrolled in the study, 23 patients had a final diagnosis of type A aortic dissection, while 113 patients had type B aortic dissection, abdominal arterial aneurysm, penetrating atherosclerotic ulcers, or intramural hematoma, and these diagnoses often overlap with each other. Two patients had mistaken coding in the discharge diagnosis, including one aortobronchial fistula and one mediastinitis. One patient had self-reported aortic dissection history without confirmed images or operational findings. Based on this anatomical and clinical rationale, we assumed that type B dissections do not involve the common carotid arteries. Therefore, patients with type B dissection were explicitly excluded during case selection. Eight of twenty-three patients with type A aortic dissection were associated with CCAD (Figure 1).

### 3.1. Patient Characteristics

The eight patients with type A dissection and concurrent CCAD were generally younger than the 15 patients without CCAD (CCAD, 56.9 [range 40–84] years versus no CCAD, 66.5 [range 41–83] years). The median age of all patients was 54 years (range 40–84). Ischemic stroke was more common in the CCAD group (CCAD, six [75.0%] versus no CCAD, four [26.7%]). Moreover, the in-hospital mortality rate was higher in the CCAD group (CCAD, three [37.5%] versus no CCAD, two [13.0%]). The in-hospital mortality rate of all type A aortic dissection patients was 21%.

A total of eight patients who had type A aortic dissection with CCAD were further studied. In addition to carotid duplex ultrasonography, the presence of CCAD was also confirmed by CT scans with and without contrast in these eight patients. The clinical features, sonographic findings, and image findings of these eight patients are shown in Table 1. There were five male patients and three female patients. In total, nine CCAD events in eight patients were identified; seven were on the right side and two were on the left side. Brain MRI revealed ischemic stroke in six patients and no ischemic stroke in two. Among the patients without stroke, patient No. 5 had asymptomatic CCA involvement, while patient No. 8 initially presented with syncope. The criteria for aortic dissection type A with asymptomatic CCAD are fulfilled if the patient does not have any ischemic stroke, transient cerebral ischemic attack, syncope, amaurosis fugax, or dizziness [14].

### 3.2. Ultrasonography Findings

**Figure 2:**
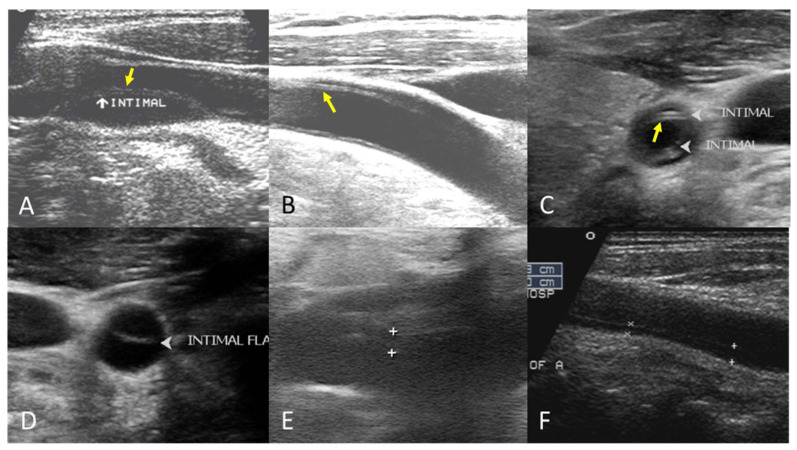
Double lumen with intimal flap with train-line pattern (**A**–**C**) and without train-line pattern (**D**–**F**). The ultrasound image was obtained using a 4× magnification setting. Yellow arrows indicate the “train line”. The train-line pattern is characterized by two closely positioned, hyperechoic parallel linear reflections overlying a thickened vessel wall. Symbols such as “×”, “+”, arrows, and arrowheads were generated by the ultrasound machine and used by sonographers to mark regions of interest (ROIs) where the intimal flap was located.

**Figure 3:**
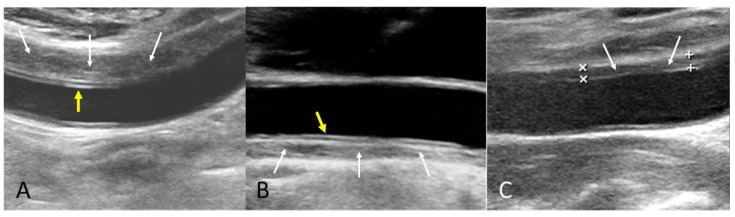
Intramural thrombus with train-line pattern (**A**,**B**) and without train-line pattern (**C**). The ultrasound image was obtained using a 4× magnification setting. Yellow arrows indicate the train line. White arrows indicate intramural thrombus. Symbols such as “×” and “+” were generated by the ultrasound machine and used by sonographers to mark ROIs.

The B-mode findings of the nine CCAs revealed six typical double lumens with intimal flaps (Figure 2) and three intramural thrombi, which are also termed thrombosed false lumen (Figure 3). Supplementary Figures S1 and S2 contain the original ultrasound images. The “train-line” pattern was recognized in three of six intimal flaps and two of three intramural thrombi. The extracranial duplex sonography revealed a “train-line” pattern characterized by a thickened wall displaying hypoechoic properties, where the boundary was composed of closely placed parallel linear reflections. Patient No. 1 corresponds to Figure 2A; patient No. 2 corresponds to Figure 2B; patient No. 3 corresponds to Figure 2C,D; patient No. 4 corresponds to Figure 2E; and patient No. 5 corresponds to Figure 2F. Patient No. 6 corresponds to Figure 3A; patient No. 7 corresponds to Figure 3B; and patient No. 8 corresponds to Figure 3C. Interestingly, patient No. 5 had bilateral CCAD, but the train-line pattern was only on the right side. Figure 2C shows the right CCA with a double lumen and a clearly visible train-line pattern, and Figure 2D displays the left CCA, which also demonstrates a double lumen, but no train-line pattern is observed. Patient No. 8 presented with a hypoechoic intramural thrombus which resembled double lumens, but it was easy to distinguish under the color Doppler imaging.

Seven of nine CCA duplex waveforms were recorded. In the waveforms of seven true lumens, three were wide systolic, one was dampened, two were biphasic, and one was second-pulsed systolic. In the waveforms of five false lumens, four were biphasic, and one was dampened and nearly occlusive. Doppler ultrasound images and pulsed Doppler waveforms are presented in Supplementary Table S1.

Follow-up carotid ultrasonography was performed in a subset of patients with the following observations: patient No. 1 underwent a follow-up carotid ultrasound six years after the initial event. The double lumen with intimal flap and the train-line pattern remained visible. Patient No. 2 did not undergo follow-up carotid ultrasonography. Patients No. 3 and No. 4 died during hospitalization and therefore did not have follow-up imaging. Patient No. 5 received a follow-up carotid ultrasound one year after the index event, which showed resolution of the double lumen and disappearance of the intimal flap. Patient No. 6 had two additional carotid ultrasound examinations on days 3 and 6 after the initial scan during hospitalization. Both studies showed a persistent intramural thrombus with a train-line pattern. Patients No. 7 and No. 8 did not undergo follow-up carotid ultrasound examinations.

We also retrospectively reviewed the carotid ultrasonographic data from the remaining 15 patients among the total 23 patients with type A aortic dissection who did not have CCAD. These patients served as a comparison group. In all 15 cases without CCAD, no train-line pattern was observed on B-mode ultrasonography. The carotid findings in these patients were limited to typical atherosclerotic plaques without features suggestive of dissection-related thrombus. This observation supports the hypothesis that the train-line pattern is specific to intramural thrombus associated with dissection and not seen in cases with only atherosclerotic changes.

## 4. Discussion

In our studies, around 33% of cases had an intramural thrombus, and 67% of cases had a double lumen with intimal flap. The “train-line” pattern is not limited to the double lumen group. It was presented in 67% of patients in the intramural thrombus group and 50% of patients in the double lumen group.

It is difficult to differentiate a dissection-related intramural thrombus from an aging-related atherosclerotic thrombus. The “train-line” pattern may help differentiate the two situations. We hypothesized that the “train-line” pattern was composed by the reflection of lumen-intima and media-intramural thrombus interfaces. Reviewing previous studies, one article also addressed the same concept that an inner intimal echo may help in distinguishing wall hematoma from the intraluminal thrombus or plaque in patients with thickened internal carotid walls [10].

The “train-line” pattern was not visualized in every case. We propose some possible explanations: different insonation angles; technical or conditional quality of ultrasound; pathologic structural difference such as intimal flap thickness or depth of vessel wall tear; the time relationship between duplex and surgery. In our study, the only two duplex ultrasonographies recorded before the operation were those of patient No. 1 and No. 6, and both patients presented with the “train-line” pattern. Further histopathology studies were required to approve the hypothesis.

Without the “train-line” pattern, we could still use traditional B mode findings to diagnose dissection in our cases. All patients in the intramural thrombus group had relatively long segments and a smooth surface of the thrombus as well as minimal atherosclerotic changes in the contralateral arterial wall. Specifically, intramural thrombus typically appears as crescentic or concentric mural thickening with a homogeneous or mildly heterogeneous hypoechoic echotexture. Its surface is usually smooth, and the lesion is located within the media or between the intima and media layers of the arterial wall [6]. Importantly, intramural thrombus is generally immobile and rarely demonstrates acoustic shadowing, as it lacks calcified components. The thrombus may cause uniform mural thickening with or without luminal narrowing. In contrast, atherosclerotic plaque is typically focal and eccentric with a heterogeneous echotexture often containing echogenic foci from fibrous or calcified elements. Its surface is irregular and may be ulcerated, and the lesion is generally confined to media [10,11]. Atherosclerotic plaques may present with instability (especially in lipid-rich soft plaques) and frequently generate posterior acoustic shadowing due to calcification. They commonly result in focal narrowing of the vessel lumen or stenosis. We summarized the distinguishing features in Table 2. But the interpretation of ultrasound was more technique-dependent, subjective and arbitrary, or sometimes equivocal [15].

In our study, patients with type A dissection and concurrent CCAD were generally younger than those without CCAD (CCAD patients: 56.9 years [range 40–84] versus non-CCAD patients: 66.5 years [range 41–83]). The incidence of ischemic stroke was higher in the CCAD group (75.0%, six patients) compared to the non-CCAD group (26.7%, four patients). Additionally, the in-hospital mortality rate was higher in the CCAD group (37.5%, three patients) compared to the non-CCAD group (13.0%, two patients). A previous retrospective study reported similar results, except for the mortality rate, which was equivalent between the two groups [16]. Therefore, cerebral malperfusion should be closely monitored when CCAD is present as an indicator [17].

A retrospective observational cohort study recruited only patients who underwent surgery for type A aortic dissection without initial neurological deficits. It reported higher rates of fatal stroke and 30-day mortality in the CCAD group than in the non-CCAD group, which was consistent with our findings. However, survival rate after one month did not differ between the two groups [14].

When compared with a larger study (Charlton-Ouw et al., 2014) [6], the results of our study are similar in median age, gender distribution, side of CCAD, percentage of double lumen and intramural thrombus. The percentage of CCAD among all aortic dissection patients is 23% in the report by Charlton-Ouw et al. and 35% in our study. Among CCAD patients, the proportion of double lumen is 65% in the previous study and 67% in ours. Thrombosed false lumen is reported as 35% in the previous report and 33% in ours. There is one discrepancy regarding the short-term prognosis, in-hospital mortality, which is 9% in the previous report and 21% in our study (Table 3).

A recent retrospective study categorized thrombosed false lumen cases into three groups based on the ratio of the thrombosed false lumen diameter to the CCA diameter: occluded (>99%), severe stenosis (70–99%), and mild stenosis (<70%) [18]. Compared with non-thrombosed false lumen cases, thrombosed cases demonstrated a significantly higher frequency of postoperative neurological deficits (23.5% vs. 43.5%) and significantly lower 5-year and 10-year survival rates but no significant difference in postoperative death. Moreover, increasing stenosis of the thrombosed false lumen in the CCA was correlated with higher in-hospital mortality, postoperative modified Rankin Scale (mRS) ≥5, postoperative neurological deficits, and 5-year mortality [18]. In our study, all patients with thrombosed false lumen were classified as experiencing mild stenosis, which may be attributable to the smaller sample size. The in-hospital mortality rate was similar between our thrombosed and non-thrombosed groups.

Emergent and proper diagnosis and treatment are key to favorable outcomes in acute aortic dissection [3,19]. The initial inaccurate diagnosis of acute aortic dissection may occur in up to 78.3% of patients [20]. Although chest pain and back pain are typical and frequent clinical presentations of aortic dissection [21], it is quite a challenge to make a timely diagnosis of aortic dissection in pain-free patients, especially when the condition is masked by ischemic stroke [22]. Up to 40% of cases who have aortic dissection are complicated with neurologic symptoms. In patients with aortic dissection, 5–15% are pain-free [5,7,23,24,25]. However, the prevalence of neurologic symptoms is even higher in patients with pain-free dissection [24]. Although studies approved the feasibility of isolated cervical artery dissection-related stroke treated with intravenous tissue plasminogen activator (tPA) [26,27,28], contemporary consensus is avoidance of thrombolytic agent for aortic dissection-related stroke [29]. Inappropriate tPA therapy may lead to fatal course [15].

Current diagnostic clues to aortic dissection include blood pressure discrepancy, hypotension, widening mediastinum on chest X-ray, dissected artery on brain MRI or enhanced CT, and double lumen with intimal flap on carotid ultrasound. Physical examinations and chest radiography are unreliable due to unsatisfactory sensitivity and specificity. CT angiography and MR angiography are sensitive to detecting aortic dissection-related stroke, but they are usually not performed routinely as initial stroke surveys [30,31]. The additional disadvantages of MRI include low availability, high cost, and consumption of time. In addition, enhanced brain CT with contrast medium brings an extra risk of nephrotoxicity.

Urgent carotid ultrasonography, which could be performed early in the emergency department, is a feasible and useful method to diagnose CCAD, which implied aortic dissection [32,33]. One case series showed that carotid ultrasonography disclosed a double lumen or an intimal flap in the CCA in 75% of the acute aortic dissection patients with stroke [34]. In many case reports, rapid carotid ultrasonography evaluation has been proven helpful to avoid possibly fatal thrombolytic treatment of acute aortic dissection with CCA extension in stroke patients [22,32,35,36,37,38,39,40]. These facts highlight the crucial role of carotid ultrasonography as an emergent bedside tool for detecting acute aortic dissection with extension to CCA in patients with acute ischemic stroke.

The advantages of carotid ultrasonography are low cost, brief examination time, and being free of radiation. It should be considered as an early diagnostic tool for detecting CCAD in these patients [41]. Disadvantages include difficulty in distinguishing dissection from atherostenosis and low sensitivity for detecting CCAD with low-grade stenosis [42]. The sensitivity of color Doppler ultrasound is 80–95% for craniocervical arterial dissection, but the sensitivity decreases with low-grade stenosis [6,10]. The “train-line” pattern, because of easy identification, is expected to increase sensitivity in diagnosing intramural hematoma irrespective of stenosis degree.

There are several inherent limitations in this study. First, our study is a retrospective design, and the findings are mainly based on observational analysis. We enrolled the patients who were diagnosed with aortic dissection and underwent carotid ultrasound during admission. Selection bias may have occurred because patients with either severe illness or excellent postoperative recovery were not included in our study due to the absence of carotid ultrasound tests. Second, the limited number of patients undergoing carotid duplex in the setting of acute type A aortic dissection with CCAD, a life-threatening emergency, represents a primary limitation of this study. The “train-line pattern” is a newly observed sonographic feature of carotid ultrasonography that, to our knowledge, has not been previously described in the literature. Our study represents the first attempt to characterize this sign based on retrospective image analysis. At present, the identification of this pattern is relatively subjective and lacks formal quantitative criteria. With a larger sample size, future studies could help develop more objective and reproducible diagnostic standards, such as measuring the length of the linear reflections, the distance between the lines, and establishing thresholds to define the presence of the pattern. These advancements could enable more standardized image interpretation and support the calculation of diagnostic performance metrics such as sensitivity, specificity, and inter-observer agreement. As a result, the generalizability of the train-line sign remains to be validated in larger prospective or multicenter cohorts. Finally, the carotid duplex is unable to visualize the entire length of the CCA, and thus dissection limited in the proximal CCA or brachiocephalic trunk would be missed by ultrasound study. For the time being, further prospective study is necessary to find out the diagnostic accuracy of the “train-line” pattern of CCA dissection.

## 5. Conclusions

Ischemic stroke is a severe and fatal complication of aortic dissection. Neurologic symptoms such as impaired consciousness and aphasia often mask underlying aortic dissection and lead to poor prognosis. Carotid duplex examination is a timely, non-invasive, low-cost, and operator-dependent technique. Double lumens were observed in two thirds of patients with CCAD resulting from aortic dissection, and intramural thrombus was observed in one third of patients. However, intramural thrombus due to CCAD stemming from aortic dissection could be mistaken for atherosclerotic plaque on ultrasonography and thus be misinterpreted. We proposed that the “train-line” pattern was composed by the reflection of lumen-intima and media-intramural thrombus interfaces. In addition to double lumen with intimal flap, the “train-line” pattern may help to distinguish intramural thrombus of dissection from atheromatous plaque.

## Figures and Tables

**Table 1:** Data on patients who had type A aortic dissection with common carotid artery dissection.

No.	Gender	Age	B Mode Findings	Train-Line	Doppler Waveform	Ischemic Stroke	Outcome
1	Female	59	Double lumen in right CCA	Present	Biphasic flow in false lumen, wide systolic wave in true lumen	Right subcortical	Survival
2	Male	42	Double lumen in left CCA	Present	Biphasic waves in false and true lumen	Right MCA territory, left basal ganglia, left parietal lobe	Survival
3	Male	47	Double lumen in both right and left CCA	Right: present Left: absent	Right: no data Left: biphasic wave in false lumen, resistive wave in true lumen	Absent	Death
4	Female	84	Double lumen in right CCA	Absent	No data	Bilateral cerebral hemispheres and left cerebellum	Death
5	Male	56	Double lumen in right CCA	Absent	Dampened and nearly occluded wave in false lumen, normal in true lumen	Bilateral watershed	Survival
6	Female	75	Intramural thrombus in right CCA	Present	Normal waveforms in true lumen	Right MCA territory	Death
7	Male	52	Intramural thrombus in right CCA	Present	No data	Right MCA territory	Survival
8	Male	40	Intramural thrombus in right CCA	Absent	Biphasic in true lumen	Absent	Survival

**Abbreviation:** CCA, common carotid artery; MCA, middle cerebral artery.

**Table 2:** Comparison of intramural thrombus and atherosclerotic plaque.

Feature	Intramural Thrombus	Atherosclerotic Plaque
Shape	Crescentic or concentric mural thickening	Focal, eccentric
Echotexture	Homogeneous or mildly heterogeneous, hypoechoic	Heterogeneous, with echogenic foci (fibrous tissue or calcification)
Surface	Smooth, layered appearance	Irregular, possibly ulcerated
Wall location	Located within media or between intima and media	Confined to media
Mobility	Generally immobile	May present with instability (especially in soft plaques)
Acoustic shadowing	Rare	Common with calcified components
Intimal flap/double lumen	May be present, suggesting dissection	Absent
Train-line pattern	Present in part of our cases	Absent in our cases

**Table 3:** Clinical features of type A aortic dissection patients.

	Present Study	Charlton-Ouw et al., 2014 [6]
Number of patients with type A aortic dissection who received carotid duplex sonography	23	87
Median age	54 years (range: 40–84)	59 years (range: 21–99)
Gender	13 male (57%), 10 female (43%)	62 male (71%), 25 female (29%)
CCAD	8 patients (35%), 9 CCAD	20 patients (23%), 26 CCAD
Side of CCAD	7 right (78%), 2 left (22%)	17 right (65%), 9 left (35%)
Double lumen	6 (67%)	17 (65%)
Thrombosed false lumen	3 (33%)	9 (35%)
Stroke	6 (75%)	5 (25%)
In-hospital mortality	5 out of 23 (21%)	8 out of 87 (9%)

## Data Availability

The datasets used and/or analyzed during the current study are available from the corresponding author on reasonable request.

## Supplementary Materials

The following supporting information can be downloaded at https://www.mdpi.com/article/10.3390/diagnostics15101297/s1, Figure S1: Original B-mode ultrasound images of CCAD patients with double lumen and intimal flap with train-line pattern (**A**–**C**) and without train-line pattern (**D**–**F**); Figure S2: Original B-mode ultrasound images of CCAD patients with intramural thrombus with train-line pattern (**A**,**B**) or without train-line pattern (**C**). Table S1: Doppler waveforms of patients No. 1 to No. 8.

## Abbreviations

The following abbreviations are used in this manuscript:

CCA	common carotid artery
CCAD	common carotid artery dissection
CT	computed tomography
MCA	middle cerebral artery
MRI	magnetic resonance imaging
mRS	modified Rankin Scale
tPA	tissue plasminogen activator
