# Supplementary material for: The Train-Line Pattern on Duplex Ultrasound Helps Differentiate Intramural Thrombus from Atheromatous Plaque in Common Carotid Artery Dissection
Source: Diagnostics (Basel). 2025 May 21;15(10):1297. doi: 10.3390/diagnostics15101297 (PMC12110014; doi:10.3390/diagnostics15101297)

Supplemental Materials

Article

The Train-Line Pattern on Duplex Ultrasound Helps Differentiate Intramural Thrombus from Atheromatous Plaque in Common Carotid Artery Dissection

Ming-Hsing Chang [1], Yen-Yu Huang [2], Fang-I Hsieh [3], Kuan-Yu Lin [1], Hsu-Ling Yeh [1], Kai-Jing Yeh [1] and Li-Ming Lien [1,4,*]

[1] Department of Neurology, Shin Kong Wu Ho-Su Memorial Hospital, Taipei 111045, Taiwan; neurotttttttt@gmail.com (M.-H.C.); alexlingy0315@hotmail.com (K.-Y.L.); mirage.yeh@gmail.com (H.-L.Y.); yehs999888@gmail.com (K.-J.Y.)
[2] Chen Sen-Feng United Clinic, Taipei 114002, Taiwan; kikilaa@hotmail.com
[3] School of Public Health, College of Public Health, Taipei Medical University, Taipei 110301, Taiwan; hsiehfangi@tmu.edu.tw
[4] College of Medicine, Taipei Medical University, Taipei 110301, Taiwan
* Correspondence: m002177@ms.skh.org.tw

List of items

Some of the original ultrasound images contained patient information at the top. To protect patient privacy, identifying details have been removed from the top portion of the images.

Figure S1: Original B-mode ultrasound images of common carotid artery dissection (CCAD) patients with double lumen and intimal flap with train-line pattern (A-C), and without train-line pattern (D-F)
Figure S2: Original B-mode ultrasound images of CCAD patients with intramural thrombus with train-line pattern (A-B) or without train-line pattern (C)
Table S1: Doppler waveforms of Patient No. 1 to No. 8.

Figure S1: Original B-mode ultrasound images of common carotid artery dissection (CCAD) patients with double lumen and intimal flap with train-line pattern (A-C), and without train-line pattern (D-F)

(A)Patient No. 1

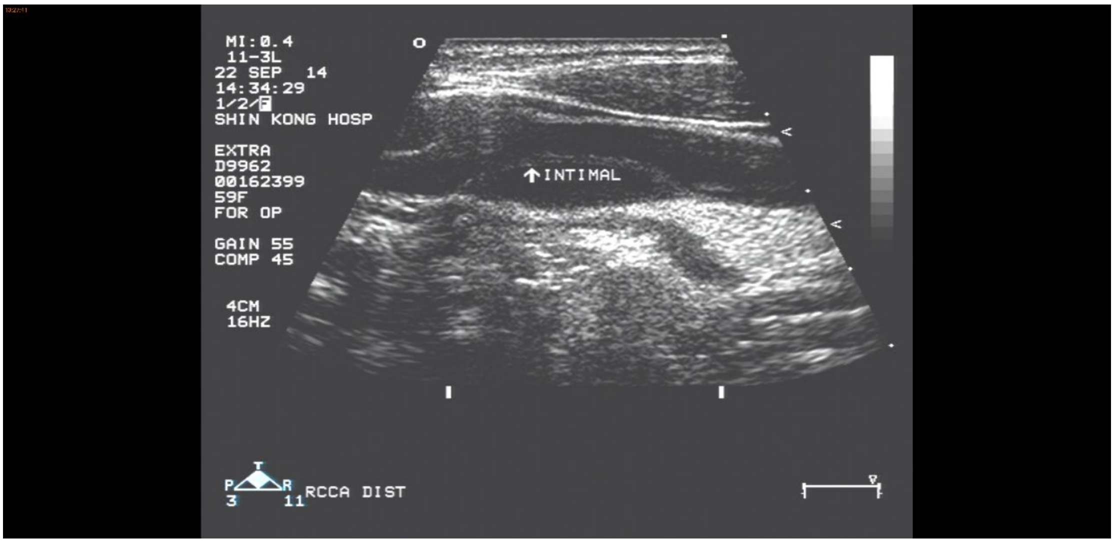

(B) Patient No. 2

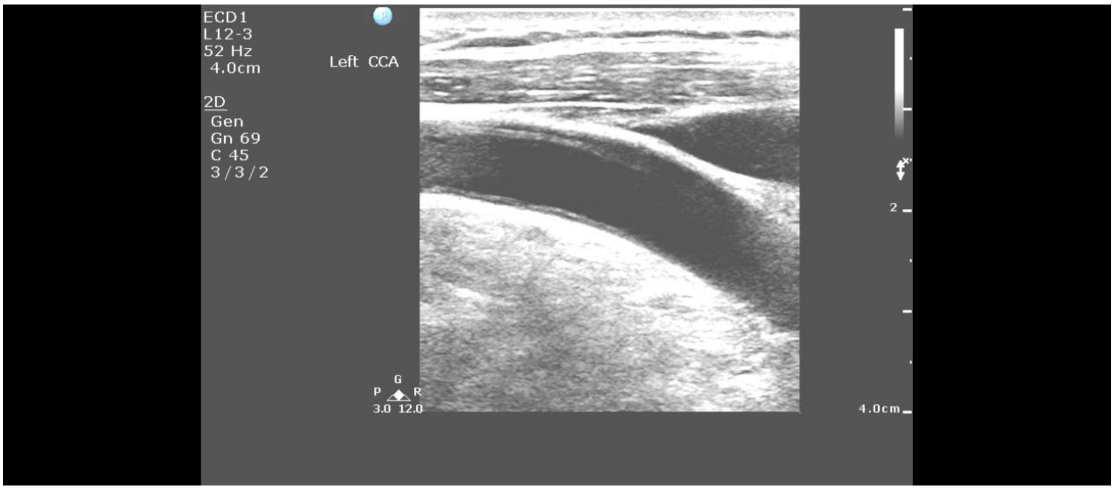

(C) Patient No. 3, right CCAD

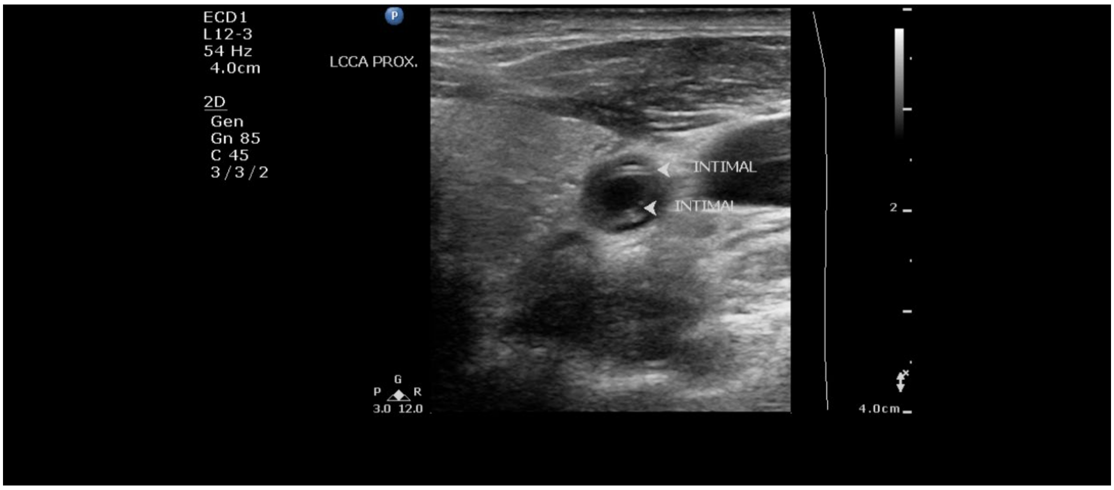

(D) Patient No. 3, left CCAD

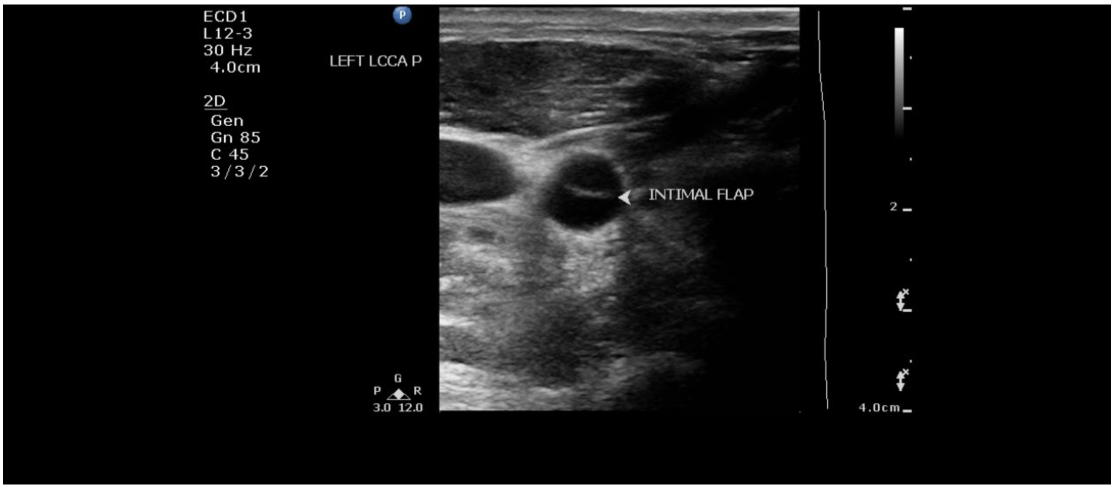

(E) Patient No. 4

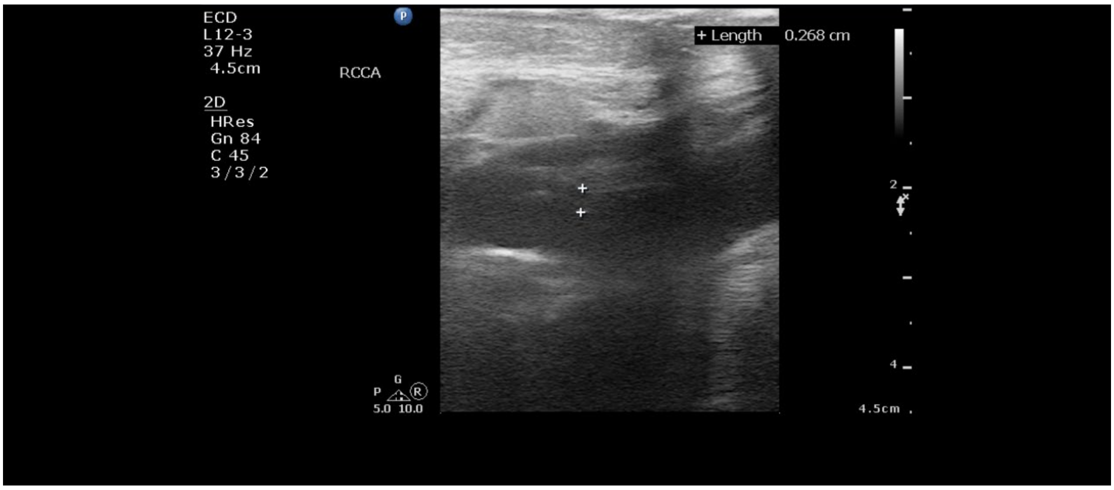

(F) Patient No. 5

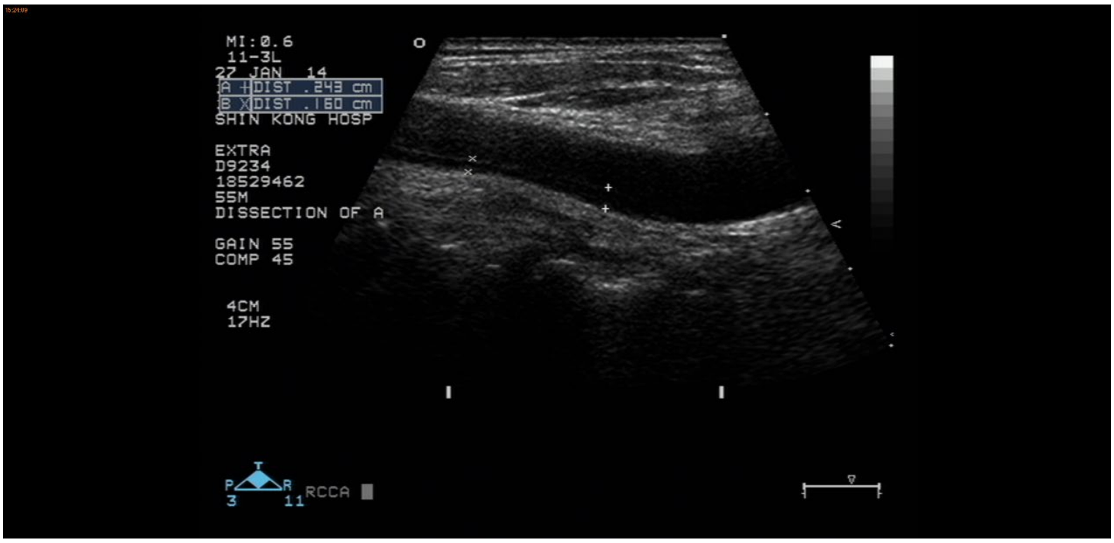

Figure S2: Original B-mode ultrasound images of CCAD patients with intramural thrombus with train-line pattern (A-B) or without train-line pattern (C)

(A)Patient No. 6

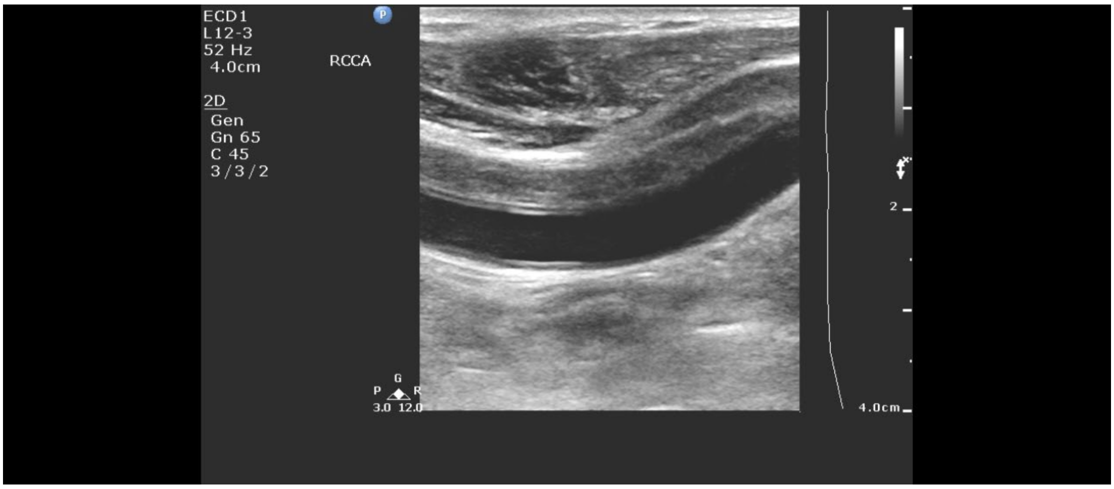

(B) Patient No. 7

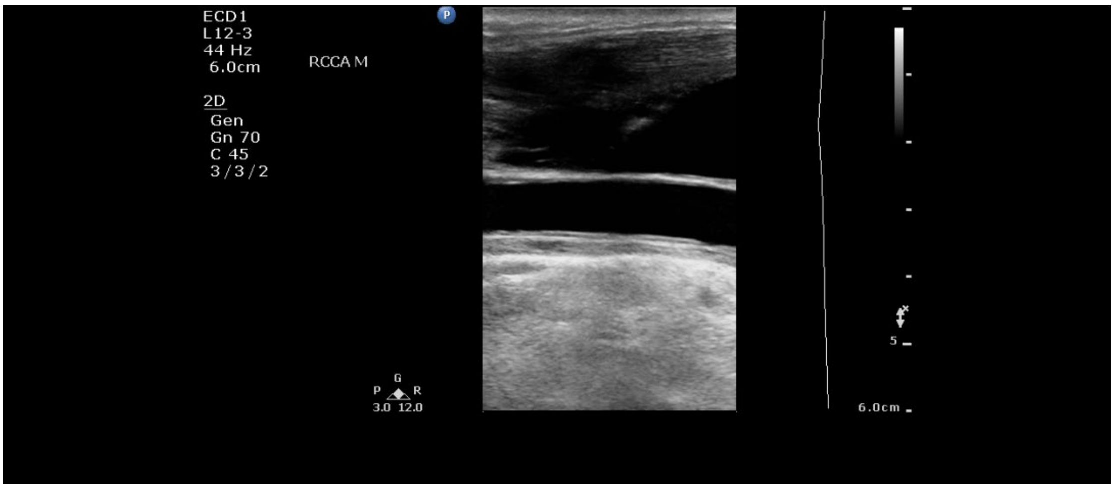

(C) Patient No. 8

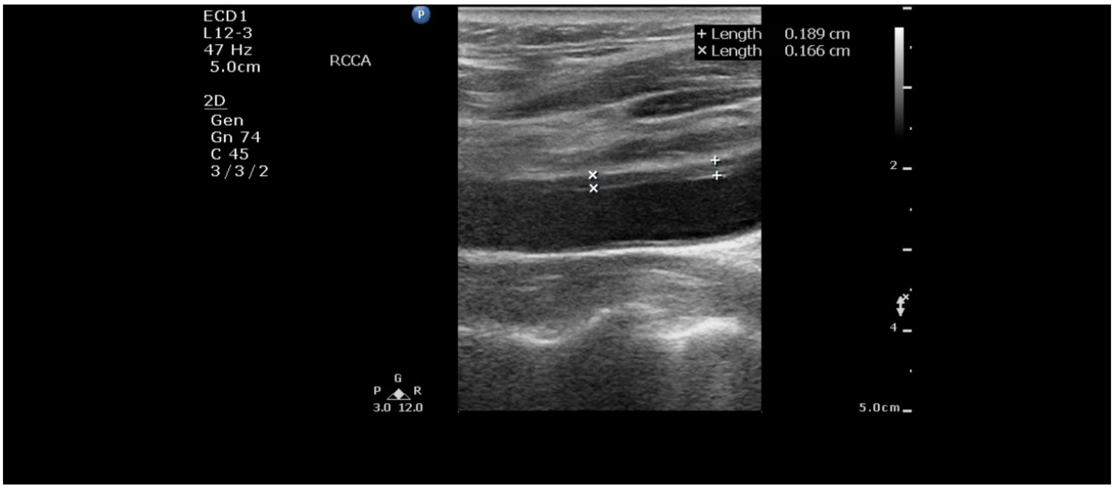

Table S1: Doppler waveforms of Patient No. 1 to No. 8.

Patient No. 1 false lumen

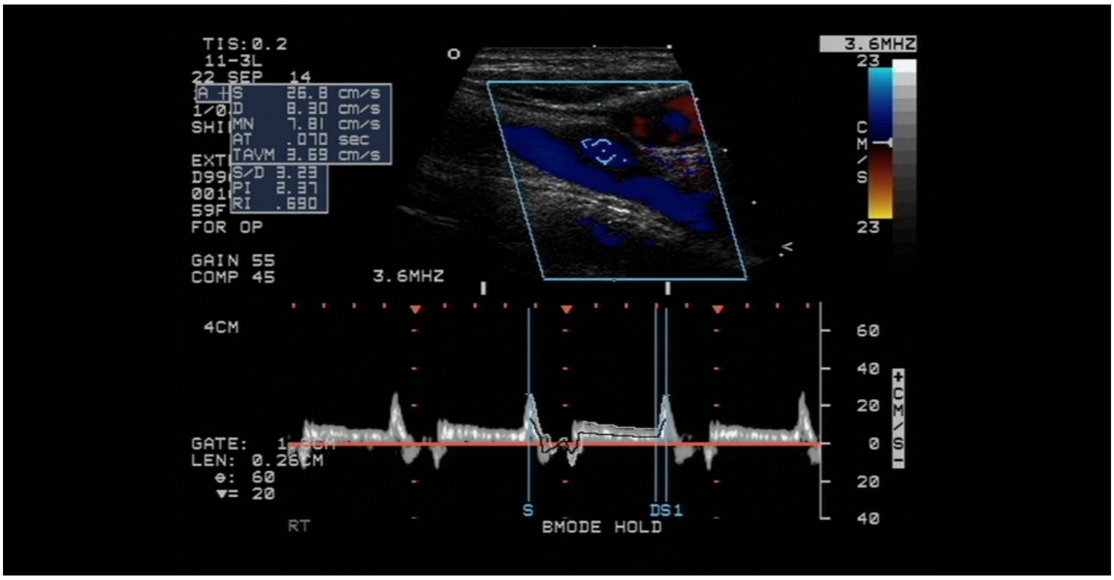

Patient No. 1 true lumen

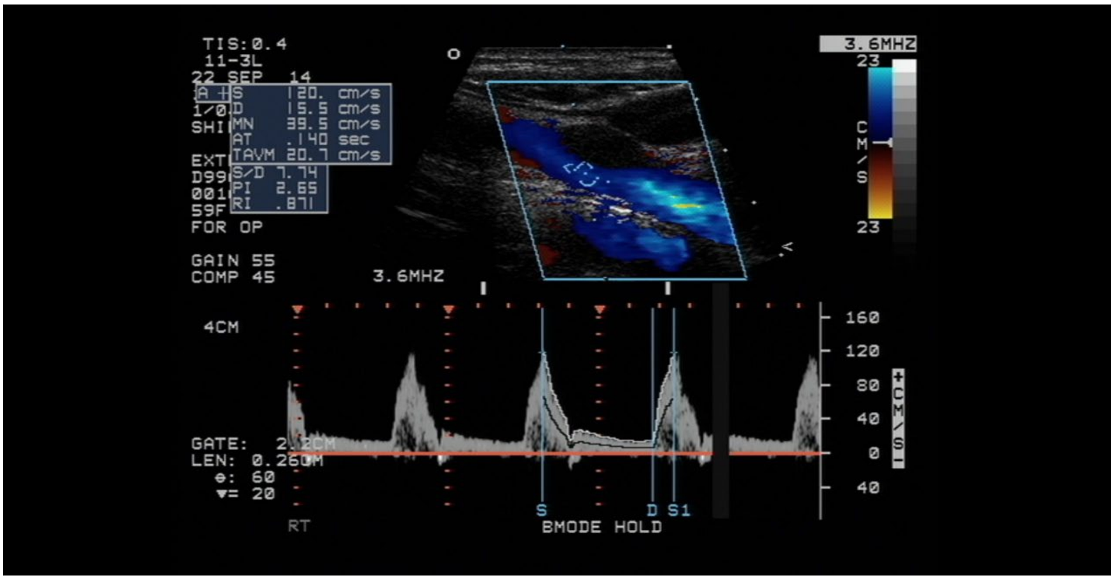

Patient No. 2 false lumen

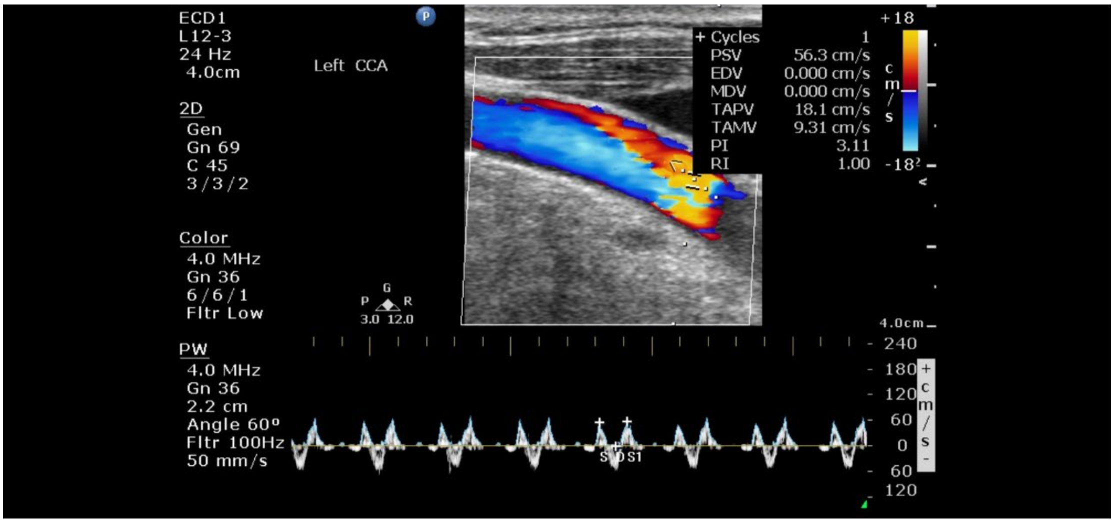

Patient No. 2 true lumen

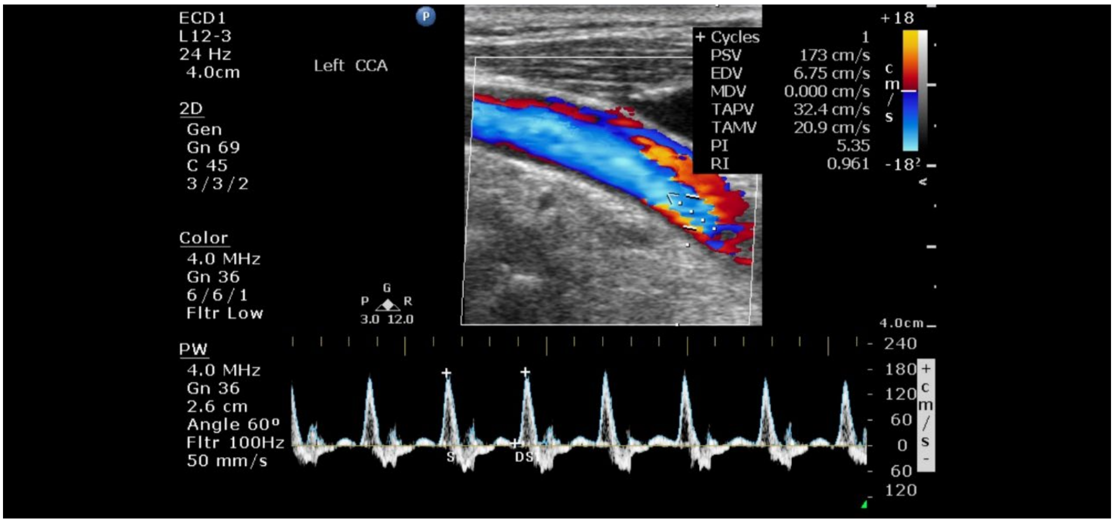

Patient No. 3 false lumen

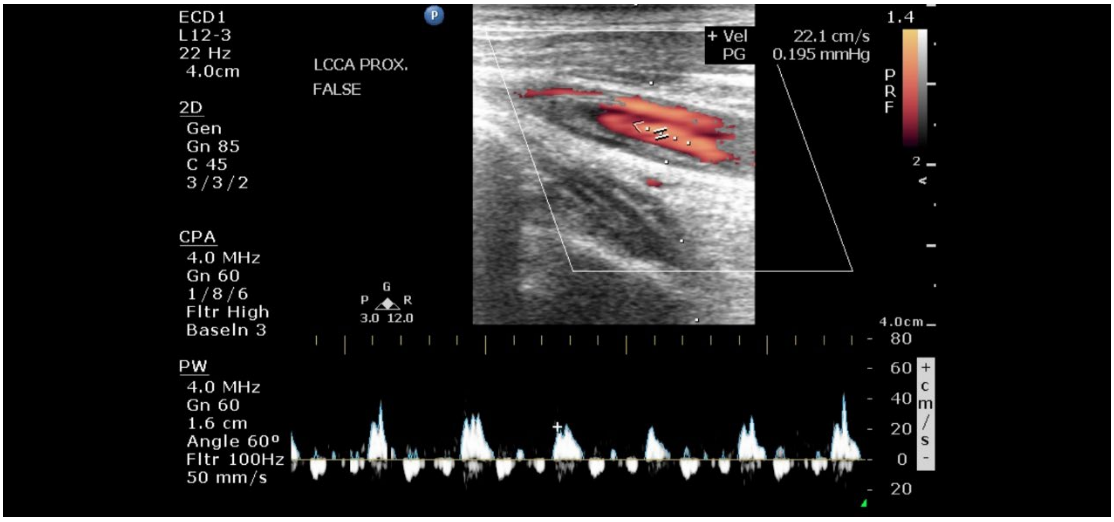

Patient No. 3 true lumen

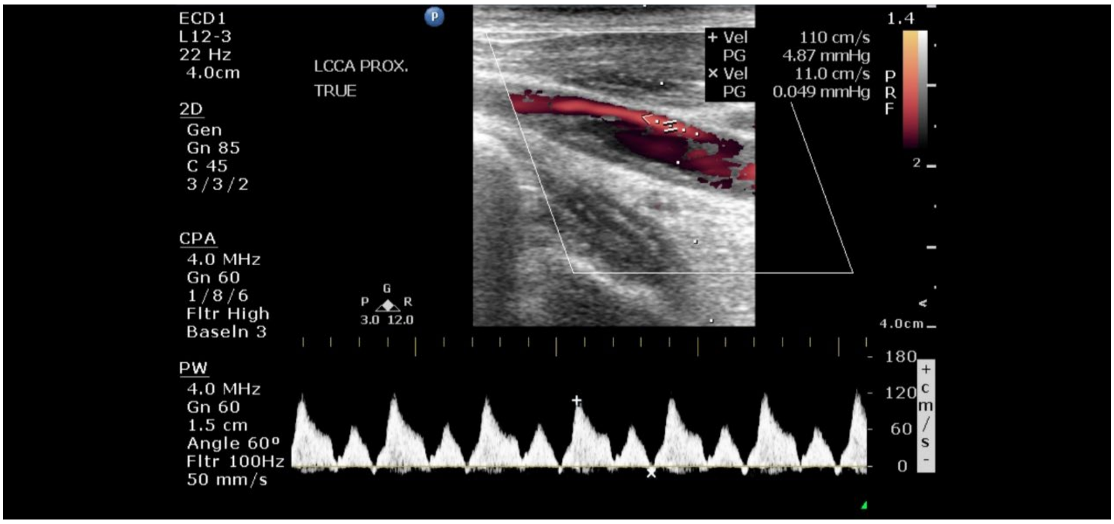

Patient No. 5 false lumen

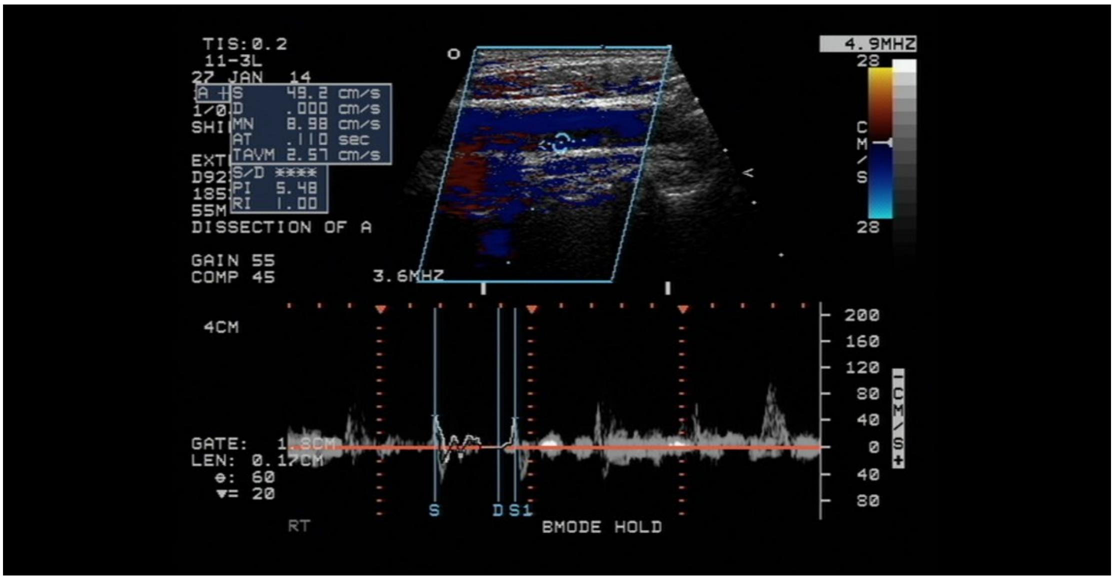

Patient No. 5 true lumen

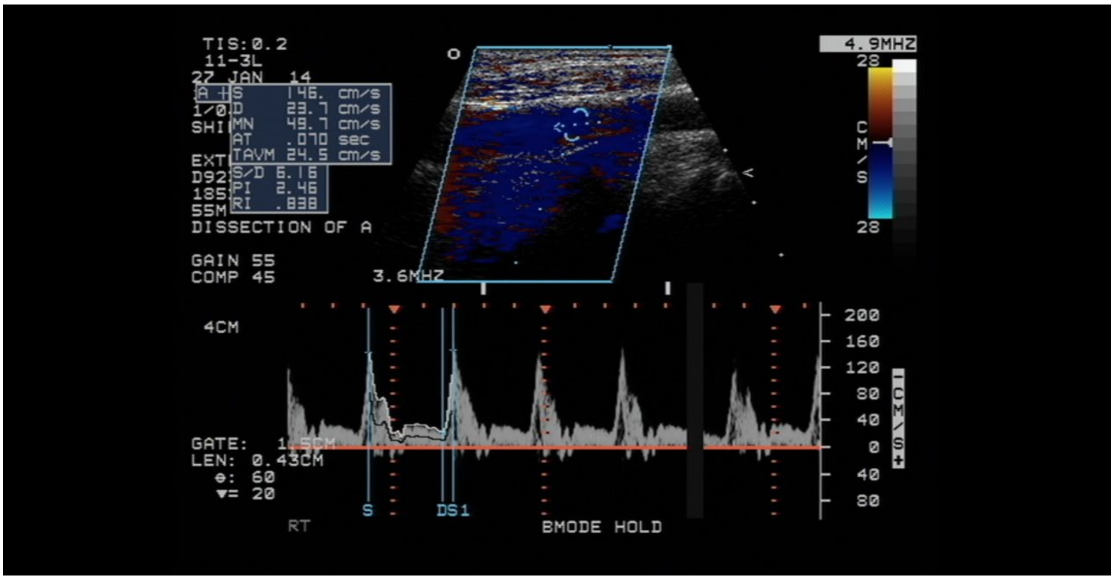

Patient No. 6 true lumen

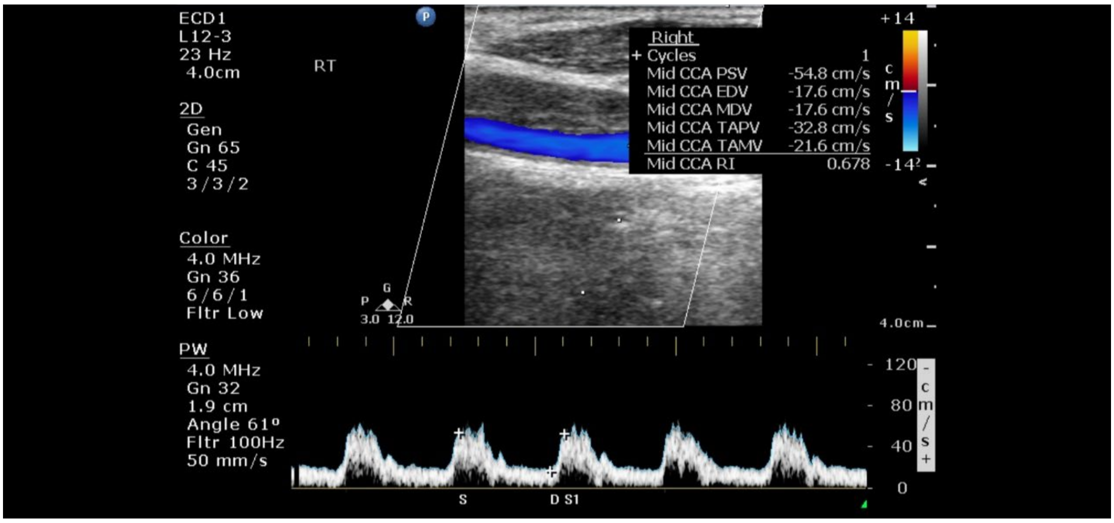

Patient No. 8 true lumen

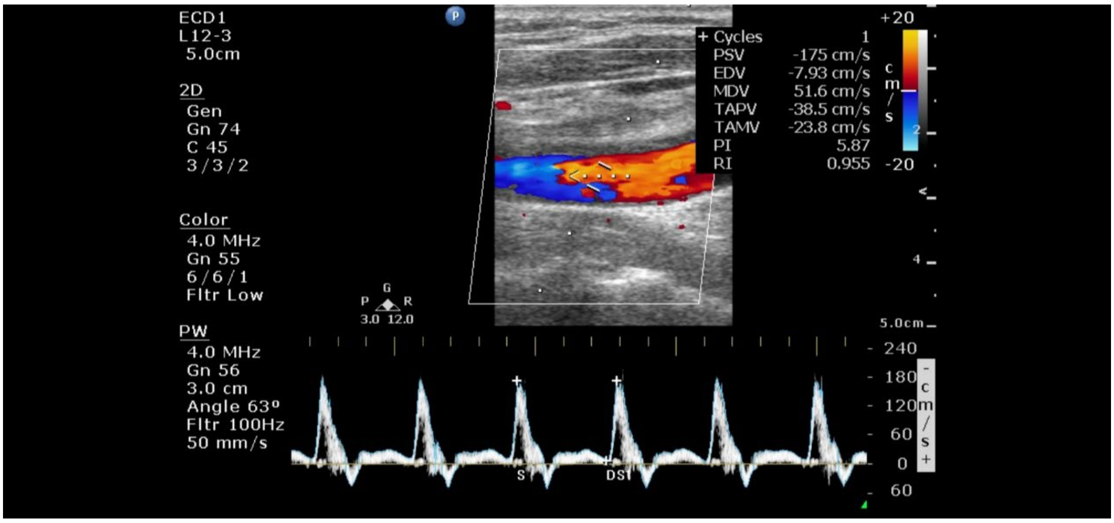

Supplement: Supplementary file 1 [file diagnostics-15-01297-s001.zip › diagnostics-3573514-supplementary.pdf]
